# Nanotechnology-based self-sterilizing surfaces and their potential in combating coronavirus disease 2019

**DOI:** 10.2217/nnm-2021-0079

**Published:** 2021-05-11

**Authors:** Parteek Prasher, Mousmee Sharma

**Affiliations:** ^1^UGC Sponsored Centre for Advanced Studies, Department of Chemistry, Guru Nanak Dev University, Amritsar, 143005, India; ^2^Department of Chemistry, University of Petroleum & Energy Studies, Energy Acres, Dehradun, 248007, India; ^3^Department of Chemistry, Uttaranchal University, Arcadia Grant, Dehradun, 248007, India

**Keywords:** COVID-19, fomites, self-sterilization

The persistence of SARS coronavirus 2 (SARS-CoV-2) RNA on surfaces in public spaces and hospitals and on personal protective equipment (PPE) serves as a potential source for the community transmission of the infection. The RNA can remain in place for a few hours to a few days under ambient conditions. The presence of moderate SARS-CoV-2 protein content in respiratory droplets discharged by infected individuals while sneezing or coughing increases the chances of virus survival on the surfaces of contaminated fomites [1]. However, the survival time of the virus on fomites depends on temperature, humidity, virus inoculum shed on the inanimate surface and nature of the surface material. After a respiratory droplet carrying SARS-CoV-2 falls on a surface, the bulk droplet evaporates, leaving behind a thin liquid film containing the virus. The droplet evaporates faster on porous surfaces than on impermeable ones [2]. Therefore, although porous materials discourage viral survival, impermeable materials, such as shiny metallic surfaces, glass and stainless steel, reportedly favor the survival of SARS-CoV-2.

## Self-cleaning materials & coronavirus disease 2019

Self-sterilizing materials possessing photosensitive characteristics offer virucidal effects by the photocatalytic triggering of reactive oxygen species (ROS) that prove detrimental to viruses [3]. Similarly, the photothermal effect offered by photosensitive materials can cause rejection of aerosolized virus particles from the contacting surface, hence providing defense against droplet infection. For example, one study found that bioinspired composite surfaces consisting of polydimethylsiloxane doped with carbon dots (PDMS-CD) underwent an 80.46% reduction in adhesive force between the surface and contaminating particles in the presence of UV radiation [4]. Irradiation of the PDMS-CD composite caused a 10°C rise in temperature, which weakened the adhesive forces with contaminating particles. Further increase in the surface temperature of the PDMS-CD composite to 50°C caused the formation of intramolecular hydrogen bonds within the composite material, resulting in a decreased number of free –OH groups on the composite surface, thereby lowering its adhesion with the contaminating particles. In addition, the rise in surface temperature of PDMS-CD composite from 30°C to 50°C led to the lowering of its surface free energy from 6.755 to 5.961 mN/m, which further explained the photothermal decrease in the adhesive forces.

The utilization of photothermal effects of nanomaterials for self-sterilization applications led to the development of photoactive antiviral masks containing a hybrid nanocoating of shellac/Cu nanoparticles with photocatalytic properties [5]. The coating of photocatalytic hybrid material on the nonwoven surgical mask improved the surface hydrophobicity and offered light-induced photothermal repulsion of aqueous droplets. Solar irradiation of the photoactive antiviral mask caused a >70°C rise in the temperature, resulting in the photocatalytic generation of free radicals that destroyed the membranes of nanosized virus-like particles with an average size of 100 nm. The photoactive antiviral mask provided defense against aerosolized virus transmission, with a rejection efficiency of 68.2%, which increased to 99.37% after irradiation with solar light. The solar light-mediated photothermal effect demonstrated by the antiviral coating developed in this study exhibited reusability due to its self-sanitization on exposure to solar radiation. This property proved particularly useful, as direct environmental disposal of the metallic nanoformulation coating on the mask could cause serious damage to the environment [6].

The severity of the coronavirus disease 2019 (COVID-19) pandemic has highlighted the importance of fabricating and developing advanced protective equipment to minimize virus transmission to frontline workers and health service providers. Karagoz *et al.* developed self-cleaning mats based on multifunctional electrospun PMMA nanofibers decorated with silver nanoparticles and ZnO nanorods [7]. The reported nanofibers, with an average diameter of 450 nm, were prepared on the nonwoven fabric by direct electrospinning to form solutions containing PMMA, ZnO nanorods and silver nanoparticles. The nanomaterial displayed considerable antibacterial properties against both Gram-positive and Gram-negative bacteria and antiviral properties against influenza virus, supporting its utility in the development of protective clothing.

Tang *et al.* evaluated the strong electrostatic interactions between anionic photosensitizers on the surface of cationic nanosized cotton fibers for the development of photoinduced nanofabrics that produce ROS on irradiation for biocidal effect [8]. The polycationic short chains in cotton nanofibers produced by self-propagation of 2-diethylaminoethyl chloride and their functionalization with anionic photosensitizers rose bengal and sodium-2-anthraquinone offered a significant (99.99%) inhibition of Gram-negative *Escherichia coli*, Gram-positive *Listeria innocua* and bacteriophage T7 within 1 h of daylight exposure. Importantly, the high content of cationic sites on the photoinduced nanofabrics improved their interactions with the anionic cell membrane of the microbes, which ameliorated the surface contact to achieve an optimal microbicidal effect. Conversely, the high content of anionic photosensitizers in photoinduced nanofabrics improved their hydrophobicity, which lowered the surface contact with the microbial cell wall, thereby compromising the biocidal efficacy of the fabric. This microbicidal efficacy presents these photoinduced nanofabrics as robust candidates for the development of PPE against microbial infections caused by nanosized aerosol droplets such as SARS-CoV-2.

For appraisal of the reusability of PPE kits coated with self-sterilizing material, Zhang *et al.* developed daylight-active, vitamin K-containing nanofibrous membranes composed of hydrophobic polyacrylonitrile and hydrophilic poly(vinyl alcohol-*co*-ethylene), which displayed resilient photoactivity for the generation of biocidal ROS [9]. A short exposure time of nanofibrous membranes to daylight or UV-A or -B radiation for less than 90 min caused >99.9% inhibition of Gram-negative *E coli*, Gram-positive *L innocua* and bacteriophage T7. In this study, the nanofibrous membranes appeared to retain their microbicidal efficacy even after repeated exposure to bacteria and viruses, hence indicating excellent reusability and supporting their application as self-sterilizing coating on PPE kits.

The emerging need for reusable self-cleaning masks has led to the investigation of whether the deposition of layers of graphene on low-melting-temperature nonwoven masks gives them superhydrophobic properties. In a study by Zhong *et al.*, these masks effectively repelled incoming aqueous droplets and demonstrated a photothermal effect when exposed to sunlight [10]. The superhydrophobicity of the material provided a defense against aerosolized droplets carrying SARS-CoV-2. When exposed to sunlight, the photothermal effect demonstrated by the deposited material raised the temperature of the material to 80°C after 100 s of exposure. By contrast, pristine surgical masks offered only a trivial photothermal effect, with a 50°C rise in temperature after 5 min of exposure to solar radiation. Reduction of the size of the graphene layer improved the photothermal performance of the material by 5°C when exposed to sunlight while retaining the original superhydrophobicity against the approaching respiratory virus-containing droplets. Therefore, the coating of layers of graphene imparted self-sterilizing properties to ordinary surgical masks, with reusability upon exposure to solar radiation, which proved economical and ecofriendly, considering the direct environmental impact of graphene-based materials [11].

Advancements in the development of self-cleaning, water-repellant fabrics gave rise to the concept of using these fabrics to filter potentially contaminated air. The development of TiO_2_–organic dye-based, water-repellent air filters by Heo *et al.* allowed the photochemical inactivation of bioaerosols in the presence of visible light [12]. The ROS generation photoinduced by the test nanomaterial caused destruction of aerosolized microbes. The visible light-activated antimicrobial air filter based on the TiO_2_–crystal violet nanocomposites created by the researchers triggered the production of ROS to achieve a 99.9% inactivation rate against bioaerosolized Gram-positive *Staphylococcus epidermidis* and *Bacillus subtilis* and Gram-negative *E coli* and *Enterobacter aerogenes*, with a considerably high filtration efficiency. The hydrophobic barrier provided by the presence of 1H,1H,2H,2H-perfluorooctyltriethoxysilane in the test material granted protection against the inhalation of aerosolized microbes present in damp air. In another study, the photocatalytic properties of TiO_2_ nanoparticle coatings were reported to inactivate SARS-CoV-2 when exposed to UV radiation [13]. The TiO_2_ nanoparticles generated ROS when briefly exposed to low-intensity UV light, which resulted in oxidative damage to the virus cells. In particular, the excitation of electrons from the valence bond to the conduction band led to the generation of an electron hole, which eventually led to the production of free radicals in the parent solution containing TiO_2_ nanoparticles. Notably, the TiO_2_ nanoparticles produced H_2_O_2_ in aqueous media, which made their virucidal activity more pronounced in the presence of moisture or humidity. The virucidal effect persisted even in dry conditions. These properties support the approach of coating surfaces with TiO_2_ nanoparticles to offer self-cleaning properties for preventing transmission of the infection [14].

Similarly, transparent surface coatings composed of Cu_2_O nanoparticle–graphene-based nanocomposites offer virucidal activity against the influenza virus by preventing its entry to host cells [15]. The nanocomposite developed in this study potentially inactivated the virus following preincubation for 30 min. The 2D graphene sheets were reported to interact with the membrane lipid layer of the virus envelope, causing agglomeration of virions on the graphene sheets and bringing them into close proximity with the Cu_2_O nanoparticles, which affected viral hemagglutinin protein structure and function. These events caused deterioration of the structural integrity of the viral envelope, eventually inhibiting the internalization of virus RNA to host cells to start the infection. The incorporation of this nanocomposite into poly(vinyl alcohol) resulted in the formation of transparent films that could be applied on fomites and inanimate surfaces to provide self-sterilizing properties. The 1- to 5-μm Cu_2_O–graphene nanocomposite incorporating 10 mM poly(vinyl alcohol) achieved 70% inhibition of the virus, offering potential application as a coating material on frequently touched surfaces to stop the spread of seasonal viral illnesses.

Nanosized covalent coatings based on quaternary benzophenone-based esters and quaternary benzophenone-based amides cross-link on surfaces when exposed to UV radiation [16]. In this study, microbes, including drug-resistant pathogens, underwent incapacitation on contacting these nanosized coatings, with 100% inhibition achieved for influenza virus. The test material performs a kill-and-release strategy against the target microbe by preventing the accumulation of dead microbial cells on the surface. The labile functionalities present in these covalent coatings are susceptible to hydrolytic degradation following which they adopt the zwitterionic structures that impart microbe-repelling properties to these coatings Furthermore, the nonleaching potency makes this desirable material for the surface coating of fomites to impart self-sterilizing properties. These antimicrobial nanocoatings offer application as photosensitive self-sterilizing material in healthcare settings in high-risk areas. Prevention of the accumulation of dead microbes by the reported nanosized covalent coatings further ensures safety against viruses, including SARS-CoV-2.

Recent advancements in the development of self-sterilizing materials has led to the discovery of superhydrophobic surfaces that prevent the settling of virus-infected aerosolized respiratory droplets. The rate of drying of respiratory droplets determines the extent of COVID-19 spread, which is fivefold greater in damp air than in dry air, as the extent of relative humidity determines the rate of evaporation of the droplets. The rate of evaporation of aerosolized droplets infected with SARS-CoV-2 increases sevenfold on elevating the humidity from 10 to 90%. The rate of evaporation also depends on the size of the aerosol droplet, which increases by roughly 2 min for a larger drop size [17]. Therefore, modifying the surface wettability and contact angle of the droplets with PPE minimizes the chances of COVID-19 infection. It has been reported that the drying time of infected respiratory droplets increases until the contact angle reaches 148°C, above which the size of the droplet and its thermophysical properties as well as initial volume become irrelevant with regard to the surface on which the drop falls [18]. The design of hydrophilic surfaces with a contact angle of 10°C reduces the drying time of infected droplets, hence playing a significant role in containing the spread of COVID-19, reducing transmission by 38%. These investigations provide useful data for designing better masks and PPE kits for combating COVID-19 transmission by droplet infection.

## Conclusion

Self-sterilizing surfaces possess the unique property of attenuating contacting microbes by inducing redox stress and by photothermal or photocatalytic destruction of the microbial cells. These materials have great potential in combating droplet infection from COVID-19 caused by infected aerosolized droplets that transmit from the patient and settle on the fomites in the vicinity. Surface coating of fomites and inanimate objects with self-cleaning antimicrobial materials serves the purpose of containing the spread of the virus by reducing its survival time or by killing the virus cell machinery, thereby preventing propagation. Self-cleaning surfaces with photothermal or photochemical properties prove effective in virus obliteration when coated on surfaces as thin films, where they display virucidal potency by creating oxidative stress, eventually killing the virus cells. Surfaces with extraordinary hydrophobicity repel aerosolized viral droplets and prove effective as coating material on masks and PPE. Considering the prevalence of so-called second waves of COVID-19 in countries around the world, self-sterilizing materials offer promise in effectively managing spread of the virus in public places and healthcare facilities, where an abundance of inanimate objects serve as sources of virus transmission. The impending self-sterilizing materials must be based on stimuli-responsive virucidal mechanisms with reduced time of action and high efficiency of virus killing. Therefore, self-sterilizing materials could prove highly effective in managing the rising number of COVID-19 cases in second waves.
